# Space-Time Adaptive Processing Based on Modified Sparse Learning via Iterative Minimization for Conformal Array Radar

**DOI:** 10.3390/s22186917

**Published:** 2022-09-13

**Authors:** Bing Ren, Tong Wang

**Affiliations:** National Laboratory of Radar Signal Processing, Xidian University, Xi’an 710071, China

**Keywords:** space-time adaptive processing, sparse learning via iterative minimization, Laplace prior, clutter suppression, conformal array

## Abstract

Space-time adaptive processing (STAP) is a well-known technique for slow-moving target detection in the clutter spreading environment. For an airborne conformal array radar, conventional STAP methods are unable to provide good performance in suppressing clutter because of the geometry-induced range-dependent clutter, non-uniform spatial steering vector, and polarization sensitivity. In this paper, a knowledge aided STAP method based on sparse learning via iterative minimization (SLIM) combined with Laplace distribution is proposed to improve the STAP performance for a conformal array. The proposed method can avoid selecting the user parameter. the proposed method constructs a dictionary matrix that is composed of the space-time steering vector by using the prior knowledge of the range cell under test (CUT) distributed in clutter ridge. Then, the estimated sparse parameters and noise power can be used to calculate a relatively accurate clutter plus noise covariance matrix (CNCM). This method could achieve superior performance of clutter suppression for a conformal array. Simulation results demonstrate the effectiveness of this method.

## 1. Introduction

The conformal array radar has many advantages, including minimal payload weight, the potential larger effective aperture, raised field of scan without the demand for cumbersome mechanical couplings, and easy integration with various sensor functions. The conformal array design also avoids signal modulation caused by antenna rotation [1]. An efficient model of the conformal array signal is a necessary prerequisite for conformal array signal processing. Compared with the traditional linear array or planar array, the conformal array has a different element pattern and normal because of the varying curvature. However, previous study assumed relatively simple element pattern and ignored the influence of the polarization and mutual coupling in clutter model.

Space-time adaptive processing (STAP) is a technique for detecting moving target in the presence of a spreading clutter background [2]. The optimal STAP weight vector is expressed as the product of the inverse of clutter plus noise covariance matrix (CNCM) and the target space-time steering vector [3]. When detecting whether the target exists in the range cell, the CNCM of the range cell is usually estimated by the training samples which are selected from the range cells adjacent to the cell under test (CUT) [4]. Traditional STAP techniques using a linear array have the acceptable performance, and the relationship between the spatial and Doppler frequencies is range-independent. Therefore, the training samples from adjacent range cells are independent and identically distributed (IID) and can estimate relatively accurate CNCM so that the STAP processor can be effectively produced to improve the output signal-interference-noise-ratio (SINR) in the test cell [5]. However, in a complex configuration, e.g., conformal array, the relationship between the spatial and Doppler frequencies is non-linear and range-dependent, and the clutter appears non-stationary over the range [6]. The Reed Mallett Brennan (RMB) rule [7] suggest that the number of IID samples should be at least twice as much as degrees of freedom to obtain a good performance. In case of conformal array, the performance of STAP degrades due to the inadequate IID samples.

In order to alleviate the influence caused by range dependence, a lot of signal processing methods have been proposed. These methods are Doppler warping (DW) [8], angle-Doppler compensation (ADC) [9], adaptive angle-Doppler compensation (A^2^ DC) [10,11], and some related methods [12,13]. These methods try to align the peaks of the clutter ridge of the training samples with that of the CUT. Unfortunately, the methods mentioned above are only available for high directivity antenna beam-patterns [14].

To resolve the drawback of inadequate IID training samples and maintain the STAP performance, many algorithms have been proposed over the last decades. The reduced-dimension (RD) methods use a dimension reducing transformation before the adaptive processing to reduce the number of the required training samples [15,16,17]. Based on the low rank character of clutter, the reduced-rank (RR) methods use the data-dependent transformation matrix to transform the clutter data into a lower dimension data [18]. Unfortunately, the training samples required by the RD and RR methods can hardly be acquired in nonstationary background. The direct data domain (DDD) STAP method proposed in Ref. [19] could avoid the problem of lacking training samples by exploiting the data of the CUT only. However, because the DDD method only utilizes partial information, the degree of freedom is reduced, and STAP performance degrades. Moreover, the DDD method has a large computational burden.

Recently, inspired by the development of sparse recovery theory [20,21], clutter sparse recovery STAP (SR-STAP) algorithms have been well developed. SR-STAP algorithms intend to recover the complex amplitudes of clutter patches by using sparse recovery theory and utilizing the clutter sparse property. By utilizing the inherent sparsity of the clutter spatial-temporal spectrum, these methods can obtain accurate CNCM with only a small number of IID training samples, even one [22,23]. A sparse representation registration-based compensation (SR-RBC) has been proposed for conformal array STAP and provide a good performance [24]. Unfortunately, these SR-STAP methods have the drawbacks like high computational burden and user parameter which is difficult to select. A STAP method based on knowledge aided sparse iterative covariance-based estimation (KASPICE-STAP) has been presented which achieve excellent performance [25]. This method utilizes the prior knowledge of the clutter ridge to construct the dictionary matrix and can estimate the relatively accurate CNCM via covariance fitting. It overcomes the drawbacks of conventional SR methods. A SR method named as sparse learning via iterative minimization (SLIM) algorithm [26,27,28] has been presented recently and it shows satisfactory performance in various applications. However, this method also has a user parameter that should be determined by incorporating the Bayesian information criterion, and the additional computational load will be brought. To overcome this problem, the Laplace distribution [29] will be utilized in SLIM.

In this paper, a new signal and clutter model which consider the influence of polarization and mutual coupling is presented. This model can be used to arbitrary array geometry and in the complex scenes. A novel KASTAP method based on modified SLIM named with KA-MSLIM-STAP is proposed here. This method overcomes the problems mentioned above. The proposed method is based on SLIM combined with Laplace distribution, which provides a more accurate expression of signals. We utilize the knowledge of the clutter ridge to obtain the ideal space-time steering vectors of clutter. These vectors will form the dictionary matrix. Then, the proposed method estimates the clutter power and noise power and calculates a relatively accurate CNCM, which can obtain good performance in the conformal array clutter suppression.

The main contributions of the paper are listed as follows:

(1) A new clutter model which considers the influence of polarization and mutual coupling is proposed. The following theoretical analysis and simulations are based on this model.
(2) The proposed method uses the accurate information of array geometry and radar system parameters to construct the CNCM based on the new received data model.
(3) The original SLIM method has a hyper-parameter that should be chosen by user. The KA-MSLIM-STAP method utilizes the Laplace distribution to avoid selecting the user parameter. It can provide an excellent performance by using the CUT data only.
(4) The proposed method is an iterative algorithm. It finds a local minimum of the cost function, but it converges rapidly. This method has lower computational complexity compared with other SR-STAP methods.

The remainder of this paper is as follows. Section 2 introduces the signal and clutter model based on the conformal array. Section 3 discusses the key theory of the proposed method and illustrate the advantages over other methods. Numerical experiments with simulated data are carried out in Section 4 to illustrate the performance of the proposed method. A summary is provided in Section 5.

*Notations*: We use boldface lower case for vectors (a), upper case for matrices (A) and the italics for scalars (a). The transpose, conjugate transpose and inverse of a matrix are represented by ${\left[\cdot \right]}^{T}$ ${\left[\cdot \right]}^{H}$ and ${\left[\cdot \right]}^{-1}$ respectively. |⋅| stands for the absolute value. ‖⋅‖ is Euclidean norm for vector and Frobenius norm for matrix. ${\left\|\cdot \right\|}_{0}$ represents the $l_{0}$ norm. ${\left\|\cdot \right\|}_{1}$ is the $l_{1}$ norm. ⊗ represents the Kronecker product. ⊙ denotes the Hadamard product. Diag(⋅) is Diagonal matrix with the vector on its diagonal. $I_{N}$ is an N×N identity matrix.

## 2. Clutter and Signal Model

### 2.1. Signal Steering Vector Model

**Figure 1:**
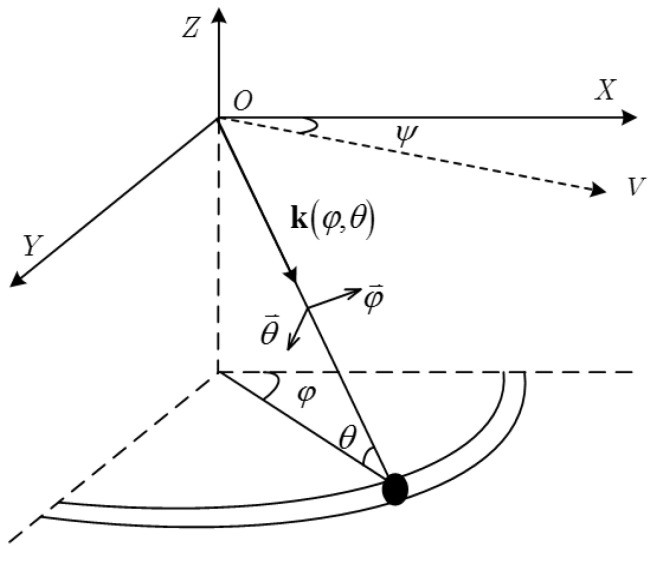
Schematic diagram of airborne radar system.

Consider a conformal array which consists of N radiating elements. As shown in Figure 1, φ and θ represent the azimuth and elevation angle of a clutter scattering point. v stands for the radar velocity.

The v is written as

(1) $$v=[v\cos\psi ,v\sin\psi ,0{]}^{T}$$

where ψ is crab angle.

The unit vector pointing to propagating direction of (φ,θ) can be represented as

(2) k(φ,θ)=[cosθcosφ,cosθsinφ,sinθ]

**Figure 2:**
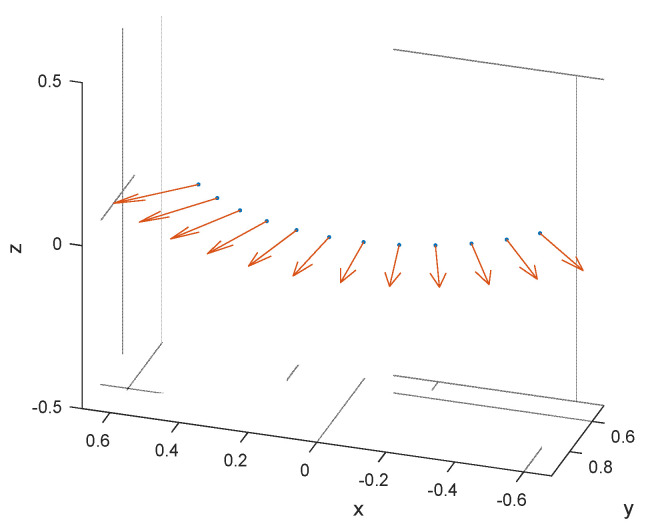
Geometry of conformal array.

A circular arc array is used here, and the configuration of conformal array is shown in Figure 2. The blue dots denote the position of the elements and red arrows represent the normal direction of elements. λ stands for the radar wavelength. The array is composed of N elements and array inter-element spacing is λ/2. The position pointing vector of the ith antenna element is $r_{i}={\left[x_{i},y_{i},z_{i}\right]}^{T}$.

The conventional normalized array manifold vector of the clutter patch from (φ,θ) can be represented as a N×1 vector

(3) $$s_{0}\left(\varphi ,\theta \right)={\left[e^{j\frac{2\pi }{\lambda }k\cdot r_{1}},e^{j\frac{2\pi }{\lambda }k\cdot r_{2}},\cdots ,e^{j\frac{2\pi }{\lambda }k\cdot r_{N}}\right]}^{T}$$

where φ is the azimuth angle and *θ* denotes elevation angle.

We design a tangential individually- polarized dipole conformal array which is composed of dipoles that are placed tangentially to circumference. Due to the different orientations, their diverse patterns sample polarization diversely, making the array polarization-sensitive.

We denote that $f_{i}$ is the ith element vector pattern. It can be decomposed into two components of orthogonal polarization, and it is shown as

(4) $$f_{i}=f_{i\vec{\varphi }}\left(\varphi ,\theta \right)\vec{\varphi }+f_{i\vec{\theta }}\left(\varphi ,\theta \right)\vec{\theta }$$

where $f_{i\vec{\varphi }}\left(\varphi ,\theta \right)$ and $f_{i\vec{\theta }}\left(\varphi ,\theta \right)$ are the φ polarized component and θ polarized component, relatively.

Because the installation direction and polarization of each element in the conformal array are different, the element vector patterns are also different in the global coordinate system.

In general, each element vector pattern is determined in their local coordinate, where $\tilde{z}$ is the normal to the surface, $\tilde{x}$ is the horizontal tangent of the carrier surface and $\tilde{y}$ is the vertical tangent of the carrier surface. Thus, we can obtain the element vector pattern ${\tilde{f}}_{i}\left(\tilde{x},\tilde{y},\tilde{z}\right)$ in the local coordinate system. In this paper, ${\tilde{f}}_{i}\left(\tilde{x},\tilde{y},\tilde{z}\right)=\left[1,0,0\right]$. This suggests that the array elements are placed along the horizontal tangent of the carrier surface. We should transform the element pattern from local coordinate to global coordinate to acquire the expression of the element pattern in the global coordinate, and the exact expression of the conformal array spatial steering vector can be further obtained. The Euler rotation matrix [30,31] is a helpful tool for the transformation.

As for the coordinate transformation $\left(x,y,z\right)\Rightarrow \left(\tilde{x},\tilde{y},\tilde{z}\right)$, the corresponding rotation matrix can be written as $T=T_{x}T_{y}T_{z}$,

where

(5) $$T_{x}=\left[\begin{array}{ccc} 1 & 0 & 0\\ 0 & \cos\alpha_{x} & -\sin\alpha_{x}\\ 0 & \sin\alpha_{x} & \cos\alpha_{x} \end{array}\right]$$

The above represents the rotation matrix with the *x*-axis as the rotating axis. $\alpha_{x}$ is the rotation angle. Counterclockwise rotation is positive.

Similarly,

(6) $$T_{y}=\left[\begin{array}{ccc} \cos\alpha_{y} & 0 & \sin\alpha_{y}\\ 0 & 1 & 0\\ -\sin\alpha_{y} & 0 & \cos\alpha_{y} \end{array}\right]$$

(7) $$T_{z}=\left[\begin{array}{ccc} \cos\alpha_{z} & -\sin\alpha_{z} & 0\\ \sin\alpha_{z} & \cos\alpha_{z} & 0\\ 0 & 0 & 1 \end{array}\right]$$

With the Euler rotation matrix, we can get

(8) $${\tilde{f}}_{i}\left(\tilde{x},\tilde{y},\tilde{z}\right)=T\cdot f_{i}\left(x,y,z\right)$$

So, we can transform the element pattern from the local coordinate to the global coordinate by

(9) $$f_{i}\left(x,y,z\right)=T^{-1}\cdot {\tilde{f}}_{i}\left(\tilde{x},\tilde{y},\tilde{z}\right)$$

With regard to the polarized components transformation from the Cartesian coordinate system to the spherical coordinate system, the following mathematical manipulations are required.

(10) $$\left[\begin{array}{c} \vec{\varphi }\\ \vec{\theta } \end{array}\right]=\left[\begin{array}{ccc} \begin{array}{c} -\sin\varphi \\ \sin\theta \cos\varphi \end{array} & \begin{array}{c} \cos\varphi \\ \sin\theta \sin\varphi \end{array} & \begin{array}{c} 0\\ -\cos\theta \end{array} \end{array}\right]\left[\begin{array}{c} \vec{x}\\ \vec{y}\\ \vec{z} \end{array}\right]$$

Then, we can obtain the $f_{i\vec{\varphi }}\left(\varphi ,\theta \right)$ and $f_{i\vec{\theta }}\left(\varphi ,\theta \right)$.

In general, for the common conformal array (e.g., cylindrical, circular, conical, and spherical array), two successive Euler rotation matrices are usually adequate. For the generalization of the conformal array signal model, three Euler rotation matrices mentioned would be used here.

Now, conformal array spatial steering vector can be expressed in the following form:

(11) $$S_{s}\left(\varphi ,\theta \right)=[F_{\varphi }\left(\varphi ,\theta \right)⊙s_{0}\left(\varphi ,\theta \right),F_{\theta }\left(\varphi ,\theta \right)⊙s_{0}\left(\varphi ,\theta \right)],S_{s}\in {\mathbb{C}}^{N\times 2}$$

where

(12) $$F_{\varphi }\left(\varphi ,\theta \right)={\left[f_{1\vec{\varphi }}\left(\varphi ,\theta \right),f_{2\vec{\varphi }}\left(\varphi ,\theta \right),\cdots ,f_{N\vec{\varphi }}\left(\varphi ,\theta \right)\right]}^{T}$$

(13) $$F_{\theta }\left(\varphi ,\theta \right)={\left[f_{1\vec{\theta }}\left(\varphi ,\theta \right),f_{2\vec{\theta }}\left(\varphi ,\theta \right),\cdots ,f_{N\vec{\theta }}\left(\varphi ,\theta \right)\right]}^{T}$$

Given the influence of mutual coupling, a mutual coupling matrix Z will be left-multiplied to form a new spatial steering vector matrix. For simplicity, we ignore the influence of mutual coupling in following section.

We assumed that array transmits a series of M pulse at a invariant pulse repetition frequency (PRF) $f_{r}$ in a coherent processing interval (CPI). The normalized Doppler steering vector can be written as a M×1 vector.

(14) $$s_{d}\left(\varphi ,\theta \right)={\left[1,e^{j\frac{2\pi }{\lambda f_{r}}2k\cdot v},\cdots ,e^{j\frac{2\pi }{\lambda f_{r}}2k\cdot v\left(M-1\right)}\right]}^{T}$$

The conformal array signal space-time steering vector can be expressed as

(15) $$S_{m}\left(\varphi ,\theta \right)=s_{d}\left(\varphi ,\theta \right)⊗S_{s}\left(\varphi ,\theta \right),S_{m}\in {\mathbb{C}}^{NM\times 2}$$

### 2.2. Conformal Array Clutter Model and Received Data Model

In clutter simulation, we make this assumption that the radar coherent processing interval (CPI) is short enough so that we can think that the velocity, RCS and polarization characteristics of the target and the RCS, polarization characteristics of the clutter patch are constant in a CPI. In this paper, we assumed that the wave received by radar array is the completely polarized wave whose polarized state does not change with time. From the above discussion, we can obtain that the conformal array can receive the polarized information of the wave. The polarization angle is μ∈[0,π/2], and the polarization phase difference is β∈[−π,π], where the definition of the polarization parameters is given in Ref. [32].

The Jones vector which can be used to express the completely polarized wave is showed as

(16) $$e_{p}=\left[\begin{array}{c} \cos\mu \\ \sin\mu e^{j\beta } \end{array}\right]$$

With the above description, the data of the clutter patch from azimuth φ and elevation θ angle $c\in {\mathbb{C}}^{NM\times 1}$ can be expressed as

(17) $$c=\varsigma \alpha S_{m}\left(\varphi ,\theta \right)\cdot e_{p}=\varsigma \alpha s\left(\varphi ,\theta \right)$$

where ς stands for a complex scalar with ς∼CN(0,1), α represents the complex amplitude of the clutter patch, and $e_{p}$ denotes the polarized state of the echo wave from clutter patch.

Therefore, the received data of the kth range cell can be represented as

(18) $$x_{k}=\sum_{l=1}^{N_{l}}\sum_{c=1}^{N_{c}}\varsigma_{l,c}\alpha_{l,c}s_{l,c}+n$$

where $N_{l}$ is the number of ambiguous range gates, $N_{c}$ indicates the number of statistically independent clutter patches of each range cell, and $s_{l,c}=S_{m}\left(\varphi_{c},\theta_{l}\right)\cdot e_{p,\left(l,c\right)}$, where $e_{p,\left(l,c\right)}$ is the polarization vector of the echo of the l,cth clutter patch, and $n\in {\mathbb{C}}^{NM\times 1}$ is the Gaussian white thermal noise vector.

The detection can be represented by following [25].

(19) $$\begin{array}{l} H_{0}:\;x=c+n\\ H_{1}:\;x=\alpha_{t}s_{t}+c+n\; \end{array}$$

where hypotheses $H_{1}$ and $H_{0}$ correspond to target presence and absence, respectively, $\alpha_{t}$ is the complex amplitude of the target and $s_{t}$ denotes the target space-time steering vector. The precise formula of the target vector can be written as

(20) $$s_{t}=S_{m}\left(\varphi_{0},\theta_{0}\right)\cdot e_{pt}$$

where $\varphi_{0}$ and $\theta_{0}$ is the azimuth angle and elevation angle of the target, respectively, $e_{pt}$ denotes the polarized state of the target.

Considering that clutter patches are mutually independent, clutter and noise are independent of each other. The CNCM $R_{k}\in {\mathbb{C}}^{NM\times NM}$ can be expressed as

(21) $$R_{k}=\sum_{l=1}^{N_{l}}\sum_{c=1}^{N_{c}}\sigma_{{}_{l,c}}^{2}s_{l,c}\cdot s_{l,c}{}^{H}+\sigma_{n}^{2}I_{NM}$$

where $\sigma_{l,c}$ is the complex amplitude of the l,cth clutter patch and $\sigma_{n}^{2}$ is the noise power.

The STAP processor can alleviate the clutter component and provide coherent gain on target by performing an inner product between the weight vector w and the data from the CUT. The well-known optimal weight vector, which maximizes the output SINR, can be written as

(22) $$w=\frac{R_{k}^{-1}s_{t}}{s_{t}^{H}R_{k}^{-1}s_{t}}$$

Because the statistics of interference environment are unknown, the CNCM is usually unknown a priori. It should be estimated by some algorithms in order to obtain an excellent performance of STAP.

## 3. The Proposed Method

### 3.1. Dictionary Matrix of Conformal Array

The range cell can be separated into $N_{k}$ cells. In this way, the snapshot can be rewritten as

(23) $$x=\sum_{k=1}^{N_{k}}\varsigma_{k}\alpha_{k}s_{k}+n=Dy+n$$

where D is a dictionary matrix, which contains steering vectors. D could be represented by

(24) $$D=\left[S_{m,1},S_{m,2},\cdots ,S_{m,N_{k}}\right]\begin{array}{cc} & {\mathbb{C}}^{NM\times 2N_{k}} \end{array}$$

$y\in {\mathbb{C}}^{2N_{k}\times 1}$ can be expressed as

(25) $$y={\left[\varsigma_{1}\alpha_{1}\cos\mu_{1},\varsigma_{1}\alpha_{1}\sin\mu_{1}e^{j\beta_{1}},\cdots ,\varsigma_{N_{k}}\alpha_{N_{k}}\cos\mu_{N_{k}},\varsigma_{N_{k}}\alpha_{N_{k}}\sin\mu_{N_{k}}e^{j\beta_{N_{k}}}\right]}^{T}$$

We note that the space-time steering vectors which is obtained from the clutter ridge are related with the array geometry and the radar system parameters. Because the elevation angle θ is constant in a certain range gate, the space-time steering vectors of clutter are determined by azimuth angle φ in essence. Therefore, we could get enough values of azimuth angle to produce the ideal space-time steering vectors corresponding to the clutter ridge of the CUT by the prior knowledge.

Here, we choose $N_{k}>NM$, and then D is an over-complete dictionary matrix which contains enough space-time steering vectors to recover the clutter accurately. y denotes sparse coefficient vector where non-zero elements represent the presence of clutter on the space-time profile. It also contains the complex amplitude and polarization parameter of the clutter. $n\in {\mathbb{C}}^{NM\times 1}$ is zero-mean Gaussian noise.

When given the range ambiguity situation, the dictionary matrix $D\in {\mathbb{C}}^{NM\times 2N_{l}N_{k}}$ can be rewritten as

(26) $$D=\left[S_{m,}{}_{1,1},S_{m,}{}_{1,2},\cdots S_{m,}{}_{1,N_{k}},\cdots ,S_{m,}{}_{N_{l},N_{k}}\right]$$

The received data from lth range gate can be represented in the following form.

(27) $$x_{l}=Dy_{l}+n_{l}$$

In the following subsection, we remark that $K=2N_{l}N_{k}$.

### 3.2. SR-STAP Model and Principle

In sparse recovery methods, we use as few atoms from dictionary matrix D as possible to represent the measurement $x_{l}$ contaminated by noise. Hence, the objective function is written as [24]

(28) $${\hat{y}}_{l}=\underset{y_{l}}{\arg\min}{\left\|y_{l}\right\|}_{0},\;\;s.t.\;\left\|x_{l}-Dy_{l}\right\|\leq \varepsilon_{1}$$

where $\varepsilon_{1}$ is a parameter that controls the error. As all known, we cannot solve (28) because it is a NP-hard problem. Hence, instead of minimizing the $l_{0}$ norm, $l_{1}$ norm has been used in the sparse represent problem [23].

(29) $${\hat{y}}_{l}=\underset{y_{l}}{\arg\min}{\left\|y_{l}\right\|}_{1},\;\;s.t.\;\left\|x_{l}-Dy_{l}\right\|\leq \varepsilon_{2}$$

where $\varepsilon_{2}$ is a user parameter.

It can be equivalent to the following optimization problem [20].

(30) $$\underset{y_{l}}{\min}\frac{1}{2}{\left\|x_{l}-Dy_{l}\right\|}_{}^{2}+\eta {\left\|y_{l}\right\|}_{1}$$

where η>0 is the user parameter. This SR method is also known as LASSO, whose disadvantages are that the user parameter is difficult to choose a compatible value and it is easily affected by the number of steering vectors. The computational complexity increases rapidly with the improvement of the size of the dictionary and the number of measurements.

### 3.3. KA-MSLIM-STAP

In this framework, to utilize the above sparsity structure, we adopt the hierarchical Bayesian model [33]. We suppose that the noise vector $n_{l}$ is a complex Gaussian random vector. It has zero mean and covariance matrix ηI. η denotes an unknown parameter. From the assumption, we obtain the following probability density functions (PDF) which can be written as

(31) $$f\left(x_{l}|y_{l},\eta \right)=\frac{1}{{\left(\pi \eta \right)}^{NM}}e^{-\frac{1}{\eta }{\left\|x_{l}-Dy_{l}\right\|}^{2}}$$

In Ref. [27],

(32) $$f\left(y_{l}\right)=\prod_{n=1}^{K}e^{-\frac{2}{q}\left({\left|y_{n,l}\right|}^{q}-1\right)}$$

where q is the hyper-parameter chosen by user. In order to making user parameter free, Ref. [27] proposed incorporating the Bayesian information criterion (BIC) to estimate q automatically. This will introduce additional computational burden. To overcome this problem, we should consider other methods.

It is reasonable that we assumed that the signal coefficients follow complex Gaussian distribution. In Ref. [28], the proposed SLIM-0 method assumed that the signal coefficients follow a complex Gaussian distribution. However, we can see from Ref. [28] that the final derived result in SLIM-0 is equivalent to the SLIM with q=0. It does not always get the optimal performance in STAP. So, we consider another distribution like Laplace distribution.

In Ref. [29], we know that Laplace prior cannot be directly applied to coefficients. To apply Laplace distribution, a hierarchical framework is modeled for the coefficient $y_{l}$.

The following prior is employed on $y_{l}$:

(33) $$p\left(y_{l}|\gamma \right)=\prod_{n=1}^{K}CN\left(y_{n,l}|0,\gamma_{n}\right)$$

where $\gamma =\left(\gamma_{1},\gamma_{2},\cdots ,\gamma_{K}\right)$. Then, model the $\gamma_{n}$ by using the following hyper priors:

(34) $$p\left(\gamma_{n}|\upsilon \right)=\Gamma \left(\gamma_{n}|1,\upsilon /2\right)=\frac{\upsilon }{2}e^{-\frac{\upsilon \gamma_{n}}{2}}$$

Combining the hierarchical Bayesian model, we can obtain:

(35) $$p\left(y_{l}|\upsilon \right)=\int p\left(y_{l}|\gamma \right)p\left(\gamma |\upsilon \right)d\gamma =\prod_{n=1}^{K}\int p\left(y_{l,n}|\gamma_{n}\right)p\left(\gamma_{n}|\upsilon \right)d\gamma_{n}=\frac{\upsilon^{K/2}}{2^{K}}e^{-\sqrt{\upsilon }\sum_{n=1}^{K}\left|y_{n,l}\right|}$$

It can be seen that the $p\left(y_{l}|\upsilon \right)$ is Laplace distribution. In this paper, we assume the signal coefficients present a Laplace distribution, that is

(36) $$f\left(y_{l}|\upsilon \right)=\frac{\upsilon^{K}}{2^{K}}e^{-\upsilon \sum_{n=1}^{K}\left|y_{n,l}\right|}$$

Then, we model υ as the following form

(37) $$f\left(\upsilon \right)\propto \frac{1}{\upsilon }$$

The estimates of $y_{l}$, υ, and η are acquired by solving a maximum a posteriori (MAP) problem as follow:

(38) $$\underset{y_{l},P,\eta }{\min}g_{MSLIM}\left(y_{l},\upsilon ,\eta \right)$$

where

(39) $$g_{MSLIM}\left(y_{l},\upsilon ,\eta \right)≜-\log\left[f\left(x_{l}|y_{l},\eta \right)\cdot f\left(y_{l}|\upsilon \right)\cdot f\left(\upsilon \right)\right]$$

From (31), (36), (37), and later discarding irrelevant constants, $g_{MSLIM}\left(y_{l},\upsilon ,\eta \right)$ can be indicated as

(40) $$g_{MSLIM}=NM\log\eta +\frac{1}{\eta }{\left\|x_{l}-Dy_{l}\right\|}^{2}-K\log\frac{\upsilon }{2}+\upsilon \sum_{n=1}^{K}\left|y_{n,l}\right|+\log\upsilon$$

The minimization problem of (38) could be resolved by utilizing the cyclic minimization (CM) method [34]. First, suppose we have obtained $y_{l}$ in the tth iteration, and the minimization problem can be written as

(41) $$\underset{\upsilon }{\min}g_{MSLIM}=-K\log\frac{\upsilon }{2}+\upsilon \sum_{n=1}^{K}\left|y_{n,l}\right|+\log\upsilon$$

We set the $\left(\partial /\partial \upsilon \right)g_{MSLIM}$ to zero, and can get

(42) $$\upsilon =\frac{K-1}{\sum_{n=1}^{K}\left|y_{n,l}\right|}$$

Then, suppose we have obtained $y_{l}$, υ and η in the tth iteration, we aim to get the $y_{l}$ and η in the t+1th iteration and the minimization problem can simplified as follows:

(43) $$\underset{y_{l},\eta }{\min}g_{MSLIM}≜NM\log\eta +\frac{1}{\eta }{\left\|x_{l}-Dy_{l}\right\|}^{2}+\upsilon \sum_{n=1}^{K}\left|y_{n,l}\right|$$

We can set the complex derivative to zero and solve it for $y_{l}$, this results in:

(44) $$\frac{1}{\eta }D^{H}Dy_{l}-\frac{1}{\eta }D^{H}x_{l}+P^{-1}y_{l}=0$$

where $P=diag\left(2\left|y_{n,l}\right|/\upsilon \right),n=1,2,\cdots ,K$.

Then, solving (44), we can get the solution

(45) $$\begin{array}{cc} y_{l} & ={\left[D^{H}D+\eta P^{-1}\right]}^{-1}D^{H}x_{l}\\ & =PD^{H}{\left[DPD^{H}+\eta I\right]}^{-1}x_{l} \end{array}$$

Similarly, setting the $\left(\partial /\partial \eta \right)g_{MSLIM}$ to zero leads to

(46) $$\eta =\frac{1}{NM}{\left\|x_{l}-Dy_{l}\right\|}^{2}$$

The solution of (38) can be obtained via repeating above iteration (42), (45), and (46) until this convergence criterion is satisfied

(47) $$\frac{\left\|y_{l}{}^{\left(t\right)}-y_{l}{}^{\left(t-1\right)}\right\|}{\left\|y_{l}{}^{\left(t\right)}\right\|}<\Delta$$

where Δ is a small, positive number. If the maximum number of iterations is attained in advance, the process suspends.

We initialize the iterative process by applying a match filter, so that $y_{n,l}{}^{\left(0\right)}=d_{n}^{H}x_{l}/{\left\|d_{n}^{}\right\|}^{2}$, for n=1,2,⋯,K, where $d_{n}$ is the nth column of D, and $\eta^{\left(0\right)}=\left(1/NM\right){\left\|x_{l}-Dy_{l}{}^{\left(0\right)}\right\|}^{2}$.

By the above iterative process, we can obtain the relatively accurate estimate of $y_{l}$ and η. The CNCM can be calculated as follows:

(48) $$R=\sum_{n=1}^{K}\left({\left\|y_{n,l}\right\|}^{2}\right)d_{n}d_{n}^{H}+\eta I$$

The weight vector could be acquired by (22). The KA-MSLIM-STAP method is summarized in Algorithm 1.

**Algorithm 1:** KA-MSLIM-STAP Method.
**Input:**


**Output:**

**Steering Vector Dictionary Matrix**
**D**

**Data**

$x_{l}$


**Space-Time Adaptive Weight Vector**

w

Step 1Initialize values of parameters as: $y_{n,l}{}^{\left(0\right)}=d_{n}^{H}x_{l}/{\left\|d_{n}^{}\right\|}^{2}$ $\eta^{\left(0\right)}=\frac{1}{NM}{\left\|x_{l}-Dy_{l}{}^{\left(0\right)}\right\|}^{2}$
Step 2Repeat the following for t=0,1,2,⋯ $\upsilon^{\left(t+1\right)}=\frac{K-1}{\sum_{n=1}^{K}\left|y_{n,l}{}^{\left(t\right)}\right|}$ $P^{\left(t+1\right)}=diag\left(2\left|y_{n,l}{}^{\left(t\right)}\right|/\upsilon^{\left(t+1\right)}\right),n=1,2,\cdots ,K$
$y_{l}{}^{\left(t+1\right)}\text{= }P^{\left(t+1\right)}D^{H}{\left[DP^{\left(t+1\right)}D^{H}+\eta^{\left(t\right)}I\right]}^{-1}x_{l}$
$\eta^{\left(t+1\right)}=\frac{1}{NM}{\left\|x_{l}-Dy_{l}{}^{\left(t+1\right)}\right\|}^{2}$ Until convergenceStep 3Calculate CNCM $R=\sum_{n=1}^{K}\left({\left\|y_{n,l}\right\|}^{2}\right)d_{n}d_{n}^{H}+\eta I$
Step 4Compute KA-MSLIM-STAP weight w


Due to the cyclic minimization character, the cost function is monotonically non-increasing in each iteration. This will indicate that the proposed method is convergent. We note that the cost function is not convex. Global optimum is not guaranteed. However, as we test numerically, excellent estimation results can be obtained, and the proposed method converges quickly. In the following simulations, this method shows excellent performance after five iterations.

The computational complexity of the methods is measured in terms of the number of multiplications. Based on the analysis above, the proposed method consists of the estimate of $y_{l}$, υ, P and η in each iteration. We can obtain that the computational complexity of the proposed method is $o\left({\left(NM\right)}^{3}+2K{\left(NM\right)}^{2}+4KNM+2K+2\right)$. The computational complexity of the KASPICE-STAP method is $o\left(2{\left(NM\right)}^{3}+2{\left(NM\right)}^{2}+2K{\left(NM\right)}^{2}+4KNM+NM+3K\right)$. We can see the proposed method has lower computational complexity.

The proposed KA-MSLIM-STAP method could acquire better performance by using the snapshot of the testing range cell only. The proposed method also possesses advantages, including low computational complexity and no need to select user parameters compared with other SR-STAP methods.

## 4. Simulation Experiments

In this section, some simulation results will show the performance of different methods with conformal array. In Figure 2, the conformal array contains 12 elements in a circular arc. We use a uniform grid here and the size is determined as $N_{k}=300$. We will verify the performance of the sample matrix inversion (SMI) method, the angle-Doppler compensation (ADC) method, the registration-based compensation with sparse representation (SR-RBC) method, the STAP method based on knowledge aided sparse iterative covariance-based estimation (KASPICE-STAP) method, the least absolute shrinkage and selection operator (LASSO) method and the proposed KA-MSLIM-STAP method with the simulated data. In this section, we consider forward-looking conformal array in the ideal condition and non-ideal condition. The range ambiguity condition is taken into account here. Then, we use the improvement factor (IF) as the measurement of performance, which can be expressed as

(49) $$\mathrm{IF}=\frac{{\left|w^{H}s\right|}^{2}}{w^{H}Rw}\frac{\mathrm{tr}\left(R\right)}{s^{H}s}$$

where R denotes the exact CNCM of the CUT.

The relevant radar parameters are given in Table 1.

### 4.1. Ideal Conditoin

The clutter data of the 300th range cell will be the input data for the spectrums.

**Figure 3:**
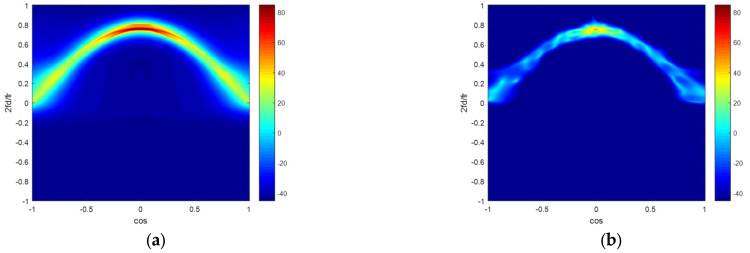

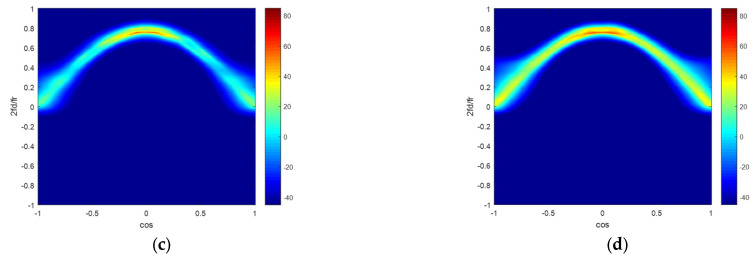
Clutter Angle-Doppler spectrums estimated by different methods in the ideal condition: (**a**) ideal CNCM; (**b**) SMI; (**c**) KASPICE-STAP; (**d**) the proposed method.

In Figure 3, there are four clutter capon spectrums figures in the ideal condition. Figure 3a–d illustrate the clutter capon spectrums estimated by the ideal CNCM, the SMI method, the KASPICE-STAP method and the proposed method. We can get that the clutter capon spectra gained by the KASPICE-STAP and the proposed method are closer to the accurate spectrum.

**Figure 4:**
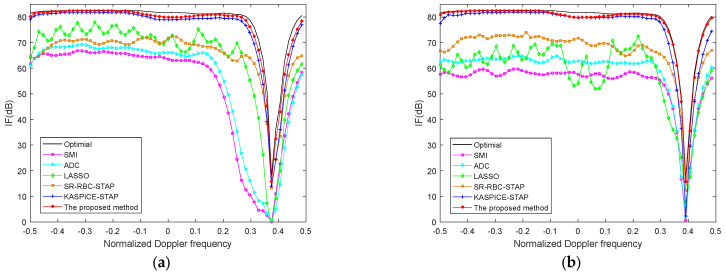
The IF versus normalized Doppler frequency with different methods in different range gates (**a**) the 300th range gate; (**b**) the 500th range gate.

Figure 4 exhibits the IF curves which are the IF versus normalized Doppler frequency with various methods in the 300th and 500th range cells. The number of training samples is chosen as 2NM in SMI, ADC, and SR-RBC methods. The number of iterations in KASPICE-STAP method is set to 5. The user parameter in the LASSO method is set to 0.001. The number of iterations in the proposed KA-MSLIM-STAP method is 5. The KA-MSLIM-STAP will show good performance.

Compared with the ADC, SMI, and SR-RBC methods, the excellent performance could be seen to emerged at the 300th and 500th range cells with the proposed method, when the number of IID training samples is inadequate. The proposed method only utilizes the testing range cell and knowledge of the clutter ridge to construct the dictionary matrix and estimate the CNCM, which can avoid the problem of inadequate IID training samples.

This subsection has exhibited the performance analysis of various methods in ideal conditions. In following subsections, we are going to probe the factors that impair the performance of these methods.

### 4.2. Model Errors

**Figure 5:**
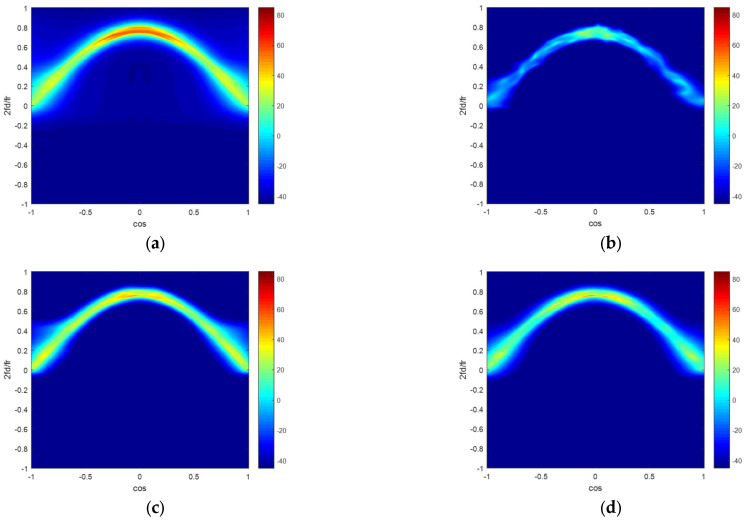
Clutter Angle-Doppler capon spectrums estimated by different methods in the non-ideal condition with model errors: (**a**) ideal CNCM; (**b**) SMI; (**c**) KASPICE-STAP; (**d**) the proposed method.

In Figure 5, we utilize four methods in the non-ideal condition with 0.5% amplitude error and 0.5° phase error. Figure 5a–d exhibit the clutter capon spectra estimated by the ideal CNCM, the SMI method, the KASPICE-STAP method, and the proposed method. We can also get that the clutter capon spectra gained by the KASPICE-STAP and the proposed method are closer to the accurate spectrum.

Because of the presence of model errors, there is a mismatch between the steering vector in dictionary matrix and the real steering vector of the clutter. This will result in the inaccurate estimation of CNCM. The model errors consist of array amplitude and phase errors.

**Figure 6:**
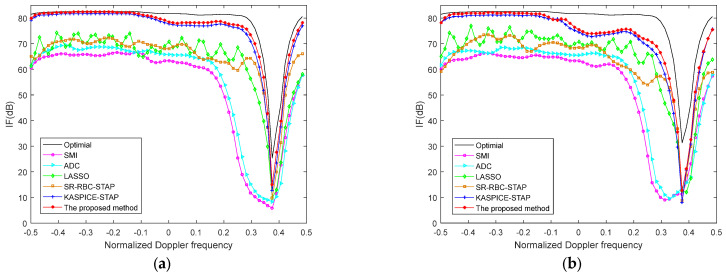
The IF versus normalized Doppler frequency with different methods (**a**) the model error are 0.5% amplitude error and 0.5° phase error; (**b**) the model error are 1.2% amplitude error and 1.2° phase error.

Figure 6 shows the IF curves with various methods with model errors, where the model errors are 0.5% amplitude error and 0.5° phase error in Figure 6a and the model errors are 1.2% amplitude error and 1.2° phase error in Figure 6b. Compared with ideal condition, the proposed method demonstrates performance degradation. The results show that the performance decrease more when model errors are large. However, we can know that the effectiveness of the proposed method is still better than other conventional methods and is excellent in terms of SR STAP methods.

### 4.3. ICM

Intrinsic clutter motion (ICM) is an issue that will exist in practice. Many factors, due to both the radar system design and the environment, may in practice cause small pulse-to-pulse fluctuations in the clutter return. The presence of ICM requires a wider clutter notch and more adaptive degrees of freedom for effective cancellation. According to Ref. [35], considering the ICM influence, the received data can be rewritten as

(50) $$x=\varsigma \alpha \left(\gamma_{t}⊙s_{d}\left(\varphi ,\theta \right)\right)⊗S_{s}\left(\varphi ,\theta \right)\cdot e_{p}$$

where $\gamma_{t}$ is the temporal covariance weight matrix.

**Figure 7:**
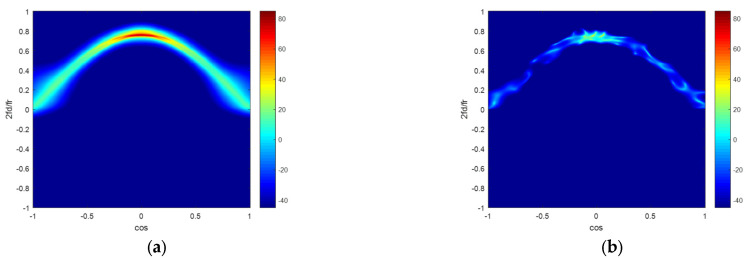

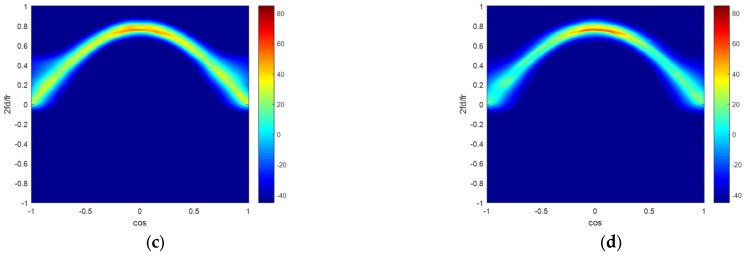
Clutter Angle-Doppler capon spectrums estimated by different methods in the non-ideal condition with ICM: (**a**) the ideal CNCM; (**b**) SMI; (**c**) KASPICE-STAP; (**d**) the proposed method.

In Figure 7, we utilize four methods in the non-ideal condition with ICM and the standard variance of clutter spectrum spread is 0.22 m/s. Figure 7a–d exhibit the clutter capon spectra estimated by the ideal CNCM, the SMI method, the KASPICE-STAP method, and the proposed method. We can get that the clutter capon spectra gained by the KASPICE-STAP and the proposed method are closer to the accurate spectrum.

**Figure 8:**
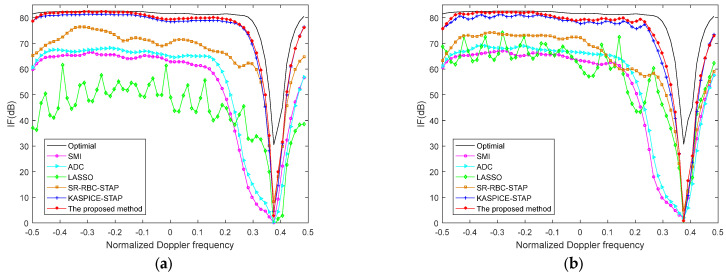
The IF versus normalized Doppler frequency with different methods (**a**) the standard variance of clutter spectrum spread is 0.22 m/s; (**b**) the standard variance of clutter spectrum spread is 0.56 m/s.

In the presence of ICM, the IF curves with different methods have been shown in Figure 8. The standard variance of the clutter spectrum spread is 0.22 m/s in Figure 8a and 0.56 m/s in Figure 8b. We can see that the proposed method still preserves desirable performance.

### 4.4. Target Detection

To further prove the performance of different methods, we use the probability of detection (PD) curves here. PD is a function of input signal-to-noise ratio (SNR) with a constant probability of false alarm $P_{fa}$. The detection curves will be acquired by the cell average constant false alarm rate (CA-CFAR) detector. The CUTs in the area of the 8–12th Doppler bins and the 301–500th range cells are selected as the 1000 runs in the Monte-Carlo experiments. We set that $P_{fa}$ is $10^{-4}$.

**Figure 9:**
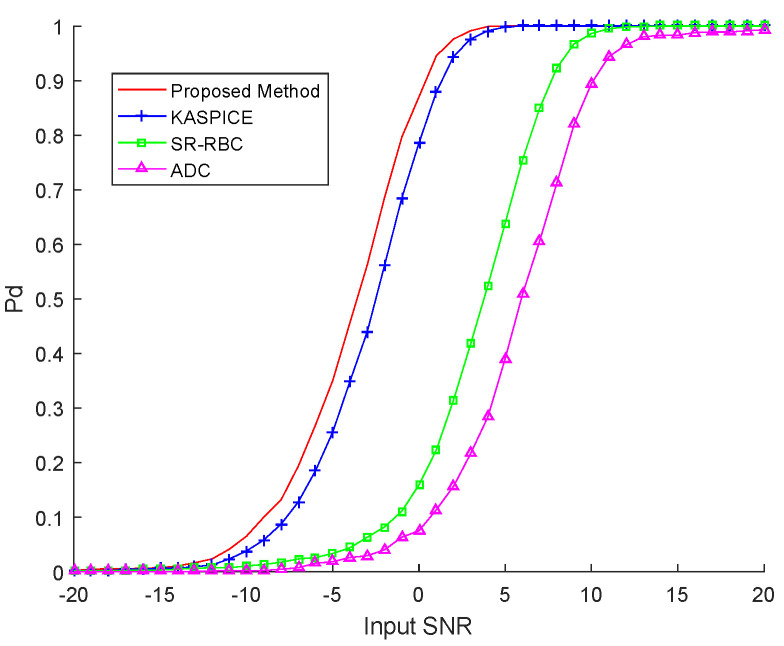
PD versus SNR with different methods.

In Figure 9, we show PD versus SNR with ADC, SR-RBC, KASPICE, and the proposed method. We can see that, compared with the detection performance of the KASPICE, there is a slight advantage of about 1 dB in that corresponding to the proposed method. Compared with the SR-RBC and ADC methods, the proposed method demonstrates great detection performance. We can conclude that the result obtained from the probability of detection performance is similar to that obtained from the performance of STAP. This can further prove the superiority of the proposed method.

## 5. Conclusions

In this paper, we have presented a novel KASTAP method based on modified sparse learning via iterative minimization named KA-MSLIM-STAP to suppress clutter for a conformal array radar. The new method reformulates the clutter model by introducing the influence of polarization and mutual coupling. Then, a new sparse learning via iterative minimization is derived with Laplace prior. The proposed method exploits the knowledge of array geometry and radar system parameters to construct the dictionary matrix and utilizes Laplace prior to avoid selecting user parameter. The effectiveness of the proposed method is verified through numerical simulations under ideal and non-ideal conditions (model errors and ICM). The results show the superiority of the proposed method in terms of clutter suppression and target detection. Moreover, we show that the proposed method has lower computational complexity compared with other SR-STAP.

For simplicity, we ignore the influence of the mutual coupling. Hence, this could be the subject of future work.

## Figures and Tables

**Table 1:** Radar system parameters.

Parameter	Value	Unit
Number of elements	12	-
Pulse number in a CPI	16	-
Wavelength	0.2	m
Distance between elements	0.1	m
Bandwidth	5	MHz
Platform height	3000	m
Pulse repetition frequency	5000	Hz
Platform velocity	200	m/s
Clutter to noise radio	60	dB
